# Light-Modulated Responses of Growth and Photosynthetic Performance to Ocean Acidification in the Model Diatom *Phaeodactylum tricornutum*

**DOI:** 10.1371/journal.pone.0096173

**Published:** 2014-05-14

**Authors:** Yahe Li, Juntian Xu, Kunshan Gao

**Affiliations:** 1 State Key Laboratory of Marine Environmental Science, Xiamen University, Xiamen Fujian, China; 2 School of Marine Science and Technology, Huaihai Institute of Technology, Lianyungang Jiangsu, China; Universidade Federal de Vicosa, Brazil

## Abstract

Ocean acidification (OA) due to atmospheric CO_2_ rise is expected to influence marine primary productivity. In order to investigate the interactive effects of OA and light changes on diatoms, we grew *Phaeodactylum tricornutum*, under ambient (390 ppmv; LC) and elevated CO_2_ (1000 ppmv; HC) conditions for 80 generations, and measured its physiological performance under different light levels (60 µmol m^−2 ^s^−1^, LL; 200 µmol m^−2 ^s^−1^, ML; 460 µmol m^−2 ^s^−1^, HL) for another 25 generations. The specific growth rate of the HC-grown cells was higher (about 12–18%) than that of the LC-grown ones, with the highest under the ML level. With increasing light levels, the effective photochemical yield of PSII (F_v_′/F_m_′) decreased, but was enhanced by the elevated CO_2_, especially under the HL level. The cells acclimated to the HC condition showed a higher recovery rate of their photochemical yield of PSII compared to the LC-grown cells. For the HC-grown cells, dissolved inorganic carbon or CO_2_ levels for half saturation of photosynthesis (K_1/2_ DIC or K_1/2_ CO_2_) increased by 11, 55 and 32%, under the LL, ML and HL levels, reflecting a light dependent down-regulation of carbon concentrating mechanisms (CCMs). The linkage between higher level of the CCMs down-regulation and higher growth rate at ML under OA supports the theory that the saved energy from CCMs down-regulation adds on to enhance the growth of the diatom.

## Introduction

Atmospheric CO_2_ concentration is expected to reach 800–1000 ppmv by the end of this century due to relentless consumptions of fossil fuels and exacerbated deforestation [1]; at the same time the oceans are taking up CO_2_ from the atmosphere at a rate of about 1 million tons per hour, leading to ocean acidification (OA) [2]. While calcifying algae are known to be sensitive to OA that decreases their calcification [3]–[7], diatoms, which account for approximately 40% of the total primary production in the oceans, show diversified or controversial responses [8]–[22]. Elevated CO_2_ concentrations are shown to enhance [8]–[14], have no effect [8], [12], [20], [21] or even inhibit [10], [12], [14], [20], [22] growth rates of diatom species. Although elevated CO_2_ in the ocean increases its availability to photosynthetic organisms, the reduced pH can influence the acid-base balance of cells [23]. In addition, the elevated CO_2_ and reduced pH levels can interact with solar radiation and temperature, showing synergistic, antagonistic or balanced effects [24]. Consequently, the mechanisms involved in the responses to OA of diatoms need to be further explored.

Diatoms in different waters experience fluctuations of light, temperature as well as changes in seawater carbonate chemistry. With the ongoing of OA, diatoms are exposed to declining pH and increased sunlight exposures in the upper mixing layer, which is shoaled due to enhanced stratification along with ocean warming [24]. It is known that phytoplankton cells exhibit different growth rates or photosynthetic performances under fluctuating light than constant light regimes [25], [26]. Therefore, it is important to see responses of diatoms to OA under different light levels or with fluctuating sunlight. Previously, we showed that OA under low or moderate levels of sunlight that fluctuates during diurnal cycles enhanced the growth of the diatoms *Phaeodactylum tricornutum*, *Skeletonema costatum* and *Thalassiosira pseudonana*, but led to growth inhibition under high (>40%) incident sunlight levels [12]. In this study, we grew the model diatom, *P. tricornutum*, in laboratory cultures under different but constant light levels (without diurnal change) to further explore the interactive effects of OA and light on its growth and photosynthetic performances.

## Materials and Methods

### Statement of Ethics

*Phaeodactylum tricornutum* Bohlin (strain CCMA 106, isolated from the South China Sea, SCS, in 2004) was obtained from the Center for Collections of Marine Bacteria and Phytoplankton of the State Key Laboratory of Marine Environmental Science, Xiamen University. No specific permits were required for using this species.

### Algal Culture Conditions

Cells of *P*. *tricornutum* were grown in 0.22 µm filtered natural seawater collected from the SCS (SEATS station: 116°E, 18°N) and enriched with Aquil medium [27]. The cultures (triplicate per CO_2_ level) were continuously aerated (350 ml min^−1^) with ambient CO_2_ level (LC, 390 ppmv) or CO_2_-enriched (HC, 1000 ppmv) air, maintained in a CO_2_ plant incubator (HP1000G-D, Ruihua Instrument and Equipment Co., Ltd., Wuhan, China), and illuminated with cool white fluorescent tubes that provided 70 µmol photons m^−2 ^s^−1^ of PAR (12L: 12D) at 20°C. Semi-continuous cultures were operated by dilution with the CO_2_-equilibrated media every 24 h, and the concentration of cells was maintained lower than 25×10^4^ cells ml^−1^, so that the seawater carbonate chemistry parameters were stable (Table 1) with pH variations <0.05 units. The pH was measured with a pH meter (Mettler Toledo DL15 Titrator, Sweden) calibrated daily with a standard National Bureau of Standards (NBS) buffer (Hanna). Other parameters of the seawater carbonate system (Table 1) were calculated using the CO2SYS software [28] taking into account the salinity, pCO_2_, pH, nutrient concentrations and temperature; the equilibrium constants K_1_ and K_2_ for carbonic acid dissociation [29] and K_B_ for boric acid [30] were referred.

**Table 1:** Chemical parameters of seawater carbonate system.

pCO_2_	pH_NBS_	DIC (µmol kg^−1^)	HCO_3_^−^ (µmol kg^−1^)	CO_3_^2−^ (µmol kg^−1^)	CO_2_ (µmol kg^−1^)	Total alkalinity (µmol kg^−1^)
LC	8.18±0.02^a^	1994.7±75.5^a^	1802.1±63.0^a^	180.0±12.5^a^	12.6^a^	2263.6±90.8^a^
HC	7.82±0.02^b^	2188.4±78.6^b^	2064.0±72.2^b^	92.1±6.4^b^	32.3^b^	2303.6±86.4^a^

Carbonate chemistry parameters of the medium for LC (ambient, 390 ppmv CO_2_) and HC (enriched, 1000 ppmv CO_2_) cultures. These parameters were averaged for 21 replicate measurements (n = 3, seven measurements for each culture). Different superscript letters represent significant difference between LC and HC.

The cultures were maintained in the above conditions for approximately 80 generations before being used in the following experiments.

### Experimental Design

The light levels were set as: photosynthesis-limited (60 µmol m^−2 ^s^−1^; LL), half-saturated (200 µmol m^−2 ^s^−1^; ML) and saturated light (460 µmol m^−2 ^s^−1^; HL) [11], with the photoperiod of 12 h d^−1^. Before and after dilution, cell concentrations were determined every 24 h with a particle count and size analyzer (Z2 Coulter, Beckman, USA). The specific growth rate was calculated as: 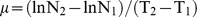, where N_1_ and N_2_ represent the cell concentrations at times T_1_ (after the dilution) and T_2_ (before the next dilution, T_2_−T_1_ = 24 h).

### Carotenoids and Chlorophyll Determination

The concentration of chlorophyll was measured by filtering the cultures onto a GF/F filter (*Φ*25 mm, Whatman), which was then extracted in 5 ml absolute methanol and maintained in darkness at least overnight at 4°C, before centrifugation (10 min at 5000 g; Universal 320R, Hettich, Germany). The absorption spectrum of the supernatant was obtained by scanning the sample from 250 to 750 nm with a scanning spectrophotometer (DU 800, Beckman, Fullerton, California, USA). Chlorophyll concentration was calculated according to [31] and that of carotenoids according to [32], as follows:
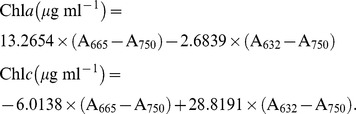


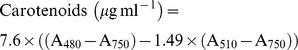



### Chlorophyll Fluorescence Measurements

Chl fluorescence was determined using a Xenon-Pulse Amplitude Modulated fluorometer (XE- PAM, Walz, Germany). To assess the photochemical responses of LC- and HC-grown cells to the changes of light, the rapid light curve (RLC), effective photochemical quantum yield (F_v_′/F_m_′) and non-photochemical quenching (NPQ) were determined. The cells harvested during the middle photoperiod were illuminated for 3 min with actinic light similar to the growth light level before measurement of RLC and F_v_′/F_m_′ to avoid effects on the photosystems caused by quasi-dark adaptation during manipulation [33]. F_v_′/F_m_′ was determined under the growth light level. The RLCs were determined at eight PAR levels (0, 29, 395, 592, 832, 1228, 1606 and 2180 µmol photons m^−2 ^s^−1^), each of which lasted for 10 s and were separated by a 0.8 s saturating white light pulse (5000 µmol photons m^−2 ^s^−1^). Parameters characteristic of the RLCs were estimated according to [34] as follows:, where a, b and c are the parameters and E is the photon flux density (µmol m^−2 ^s^−1^). The maximal rate of relative electron transport (rETR_max_), the light harvesting efficiency (α) and the initial light saturation point (E_k_) were calculated according to [34] from the fitted RLC: 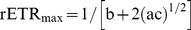; 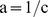; 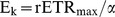. After 15 min dark adaptation, fluorescence induction curves were measured with the actinic light of 1228 µmol m^−2 ^s^−1^. The NPQ was calculated as: 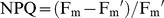, where F_m_ represents the maximum fluorescence yield after dark adaptation (15 min) and F_m_′, the maximum fluorescence yield determined at the actinic light levels.

In order to determine the effects of the dim light on the recovery of the PSII, the changes of F_v_/F_m_ were determined when the cells were transferred to the dark or dim light (10 µmol m^−2 ^s^−1^) conditions.

### Assessment of Photosynthetic Affinity for Dissolved Inorganic Carbon (DIC)

We applied Chl fluorescence technique to obtain the relationship of photosynthesis with DIC levels according to Wu et al. [11]. Briefly, the cells were harvested, washed with, and re-suspended in DIC-free seawater medium buffered with 20 mM Tris (pH 8.18) [35] at a final density of ∼3×10^4^ cell ml^−1^, and then were incubated at 400 µmol m^−2 ^s^−1^ for 15 min to exhaust intracellular DIC before sodium bicarbonate solution was injected to obtain different DIC concentrations between 0–2200 µmol L^−1^. The relative electron transport (rETR) was determined as mentioned above, and the K_1/2_ (reciprocal of photosynthetic affinity) values for DIC or CO_2_ were calculated using the Michaelis-Menten equation.

### Data Analysis

The two way ANOVA (Tukey-test) was used to establish differences among the treatments, and the significance level was set at *p*<0.05.

## Results

### Carbonate Chemistry System

The pH levels in the LC and HC cultures were 8.18 (±0.02) and 7.82 (±0.02). In the HC cultures, DIC, HCO_3_^−^ and CO_2_ levels were significantly higher (by 9.7%, 14.5% and 156.3%, respectively) and the CO_3_^2−^ level was lowered by 48.8%. There was no significant difference in total alkalinity between the LC and HC cultures (Table 1).

### Growth Rate and Photosynthetic Pigments

**Figure 1:**
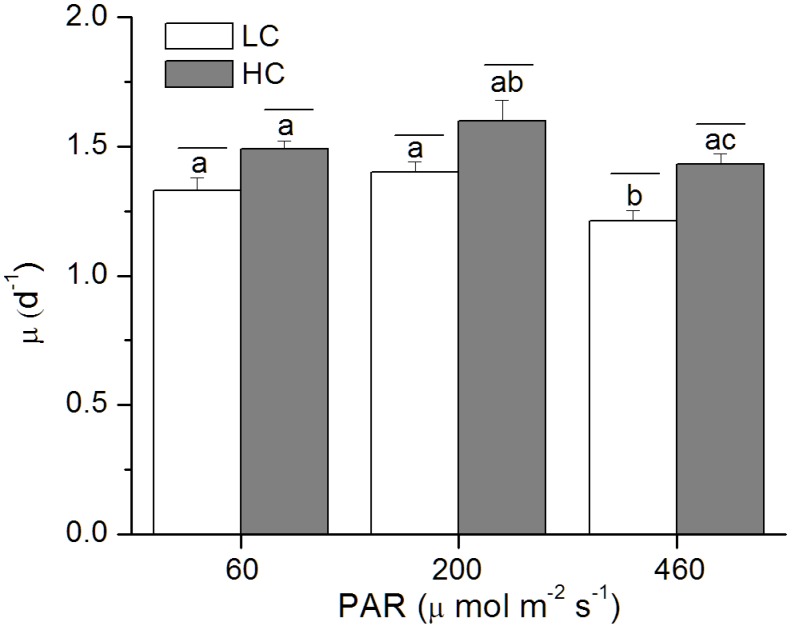
Specific growth rates of *P. tricornutum*. Growth rates of *P. tricornutum* cells grown under the LC (390 ppmv, pH 8.18) and HC (1000 ppmv, pH 7.82) and then both acclimated to different light levels (60, 200 and 460 µmol m^−2 ^s^−1^) for 25–36 generations. Values are means ± SD, n = 3. The short-lines above the histogram bars indicate significant difference between LC and HC, and the different letters indicate significant differences among the light treatments within the HC- or LC-grown cells at *p*<0.05.

**Figure 2:**
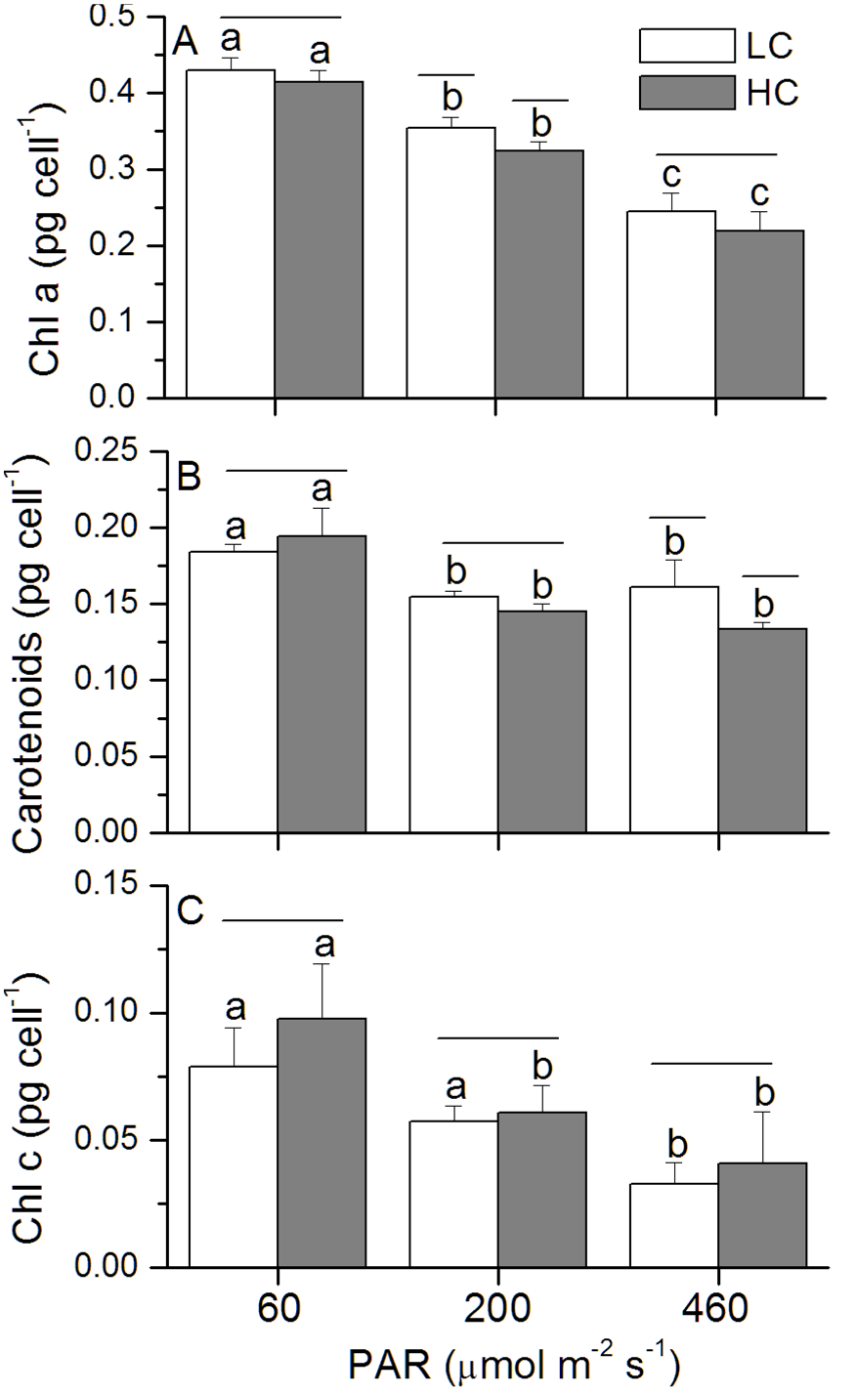
Pigmentation of *P. tricornutum*. Chl α (A), Carotenoids (B) and Chl *c* (C) of *P. tricornutum* cells grown under the LC (390 ppmv, pH 8.18) and HC (1000 ppmv, pH 7.82) and then both acclimated to different light levels (60, 200 and 460 µmol m^−2 ^s^−1^) for 25–36 generations. Values are means ± SD, n = 3. The short-lines above the histogram bars indicate significant difference between LC and HC, and the different letters indicate significant differences among the light treatments within the HC- or LC-grown cells at *p*<0.05.

Compared to the LC-grown cells, elevated CO_2_ enhanced the specific growth rate by 12, 14 and 18% under the low, medium and high light levels (Fig. 1). The growth rate was highest at the medium but lower at the low and high light levels (Fig. 1). Chl *a* concentration per cell sharply declined (*p*<0.01) with increased light levels, with a significant difference between LC- and HC-grown cells (*p* = 0.02; Fig. 2A) under the medium light level and insignificant effects under the low (*p* = 0.18) and high light levels (*p* = 0.22) (Fig. 2A). For the content of carotenoids, significant difference between the two CO_2_ levels was observed under the HL level (*p* = 0.01; Fig. 2B). Chl *c* content increased in the HC-grown cells, although the difference was only marginally significant (*p* = 0.08–0.71) (Fig. 2C). HL-grown cells showed the highest ratio between the carotenoids and Chl *a*, but the lowest ratio of Chl *c* to Chl *a*, and the HC-grown cells had lager values for the ratio of Chl *c* to Chl *a*, although the difference between the CO_2_ treatment was not significant (Table 2).

**Table 2:** Ratios between carotenoids, Chl *c* and Chl *a* concentrations of *P. tricornutum*.

PAR (µmol m^−2 ^s^−1^)	Carotenoids/Chl *a*		Chl *c*/Chl *a*	
	LC	HC	LC	HC
60	0.43±0.027^a^	0.47±0.048^a^	0.18±0.030^a^	0.23±0.046^ab^
200	0.44±0.006^a^	0.45±0.029^a^	0.16±0.008^ac^	0.19±0.027^a^
460	0.65±0.039^b^	0.61±0.083^b^	0.13±0.015^ac^	0.18±0.044^a^

The ratio between carotenoids, Chl *c* and Chl *a* concentrations of *P. tricornutum* cells grown under the LC (390 ppmv, pH 8.18) and HC (1000 ppmv, pH 7.82) and then both acclimated to different light levels (60, 200 and 460 µmol m^−2 ^s^−1^) for 25–36 generations. Values are means ± SD, n = 3. Different superscript letters indicate significant differences among different treatments within the ratio between carotenoids or Chl *c* and Chl *a* at *p*<0.05.

### Photochemical and Non-photochemical Responses

**Figure 3:**
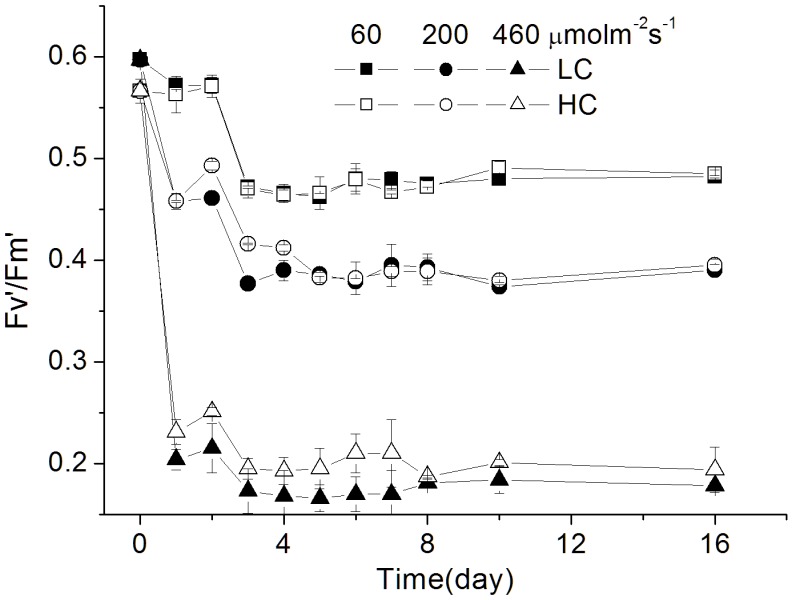
The effective photochemical yield (F_v_ ′**/F_m_**′**) of** ***P. tricornutum*** **.** The effective photochemical yield (F_v_′/F_m_′) of *P. tricornutum* cells grown under the LC (390 ppmv, pH 8.18) and HC (1000 ppmv, pH 7.82) and then both acclimated to different light levels (60, 200 and 460 µmol m^−2 ^s^−1^) for different generations (from 0 to 36). Values are means ± SD, n = 3.

The effective quantum yield of the PSII, F_v_′/F_m_′, showed constant values, at 0.47, 0.40, 0.18 under the low, medium and high light levels, and was significantly enhanced by elevated CO_2_ under the HL condition (*p*<0.01; Fig. 3).

In views of rapid light curves (RLC), the saturation PAR level (E_k_) significantly increased with the increase of growth light levels (*p*<0.01), but decreased with the acclimation time from day 1 to day 16, while the apparent photosynthetic efficiency (α) showed the opposite trends (*p*<0.01) (Table 3). After acclimation to different light levels for 25–36 generations, the apparent light use efficiency (α) was enhanced in the HC-grown cells under the low (*p* = 0.004), medium (*p* = 0.001) and high (*p* = 0.02) light levels, but the maximal electron transport (rETR_max_) showed no significant variations between the LC and HC cultures (Table 3).

**Table 3:** The fitted parameters derived from the rapid light curves of *P. tricornutum*.

Generations and parameters		LC			HC		
		60	200	460	60	200	460
	E_k_	370±15.7^a^	672±9.9^a^	1313±146.7^a^*	426±72.1^a^	680±38.8^a^	1157±76.4^a^*
1–2	rETR_max_	105±1.1^a^	133±0.3^a^	127±7.9^a^*	113±7.5^a^	133±7.4^a^	138±3.8^a^*
	α	0.28±0.005^a^	0.20±0.003^a^	0.10±0.017^a^	0.27±0.027^a^	0.20±0.001^a^	0.12±0.011^a^
	E_k_	314±18.7^a^	629±12.7^a^*	1167±28.1^b^*	342±9.1^a^	654±16.1^a^*	1076±82.1^ab^*
12–18	rETR_max_	77±5.1^b^	102±1.6^b^	107±8.7^b^	81±2.6^b^	117±0.4^b^	118±5.4^b^
	α	0.24±0.001^b^	0.16±0.001^b^	0.10±0.005^a^	0.24±0.014^b^	0.18±0.005^a^	0.11±0.007^a^
	E_k_	261±6.4^b^	546±17.2^b^	1060±41.0^c^	248±20.0^b^	481±1.3^b^	982±79.8^b^
25–36	rETR_max_	81±0.6^b^	115±2.9^c^	118±4.8^a^	82±0.9^b^	113±0.9^b^	127±4.5^b^
	α	0.30±0.010^a^*	0.20±0.001^a^*	0.10±0.001^a^*	0.33±0.031^c^*	0.24±0.002^b^*	0.13±0.019^a^*

The fitted parameters derived from the rapid light curves of *P. tricornutum* cells grown under the LC (390 ppmv, pH 8.18) and HC (1000 ppmv, pH 7.82) and then both acclimated to different light levels (60, 200 and 460 µmol m^−2 ^s^−1^) for different generations (from 1 to 36 generations): E_k_, the initial light saturation point (µmol m^−2 ^s^−1^); rETR_max_, the maximal rate of relative electron transport; α, the apparent light use efficiency. Values are means ± SD, n = 3 (triplicate cultures). Different superscript letters represent significant among the different generations within the LC or HC-grown cells and the asterisks indicate significant difference between LC and HC at *p*<0.05.

**Figure 4:**
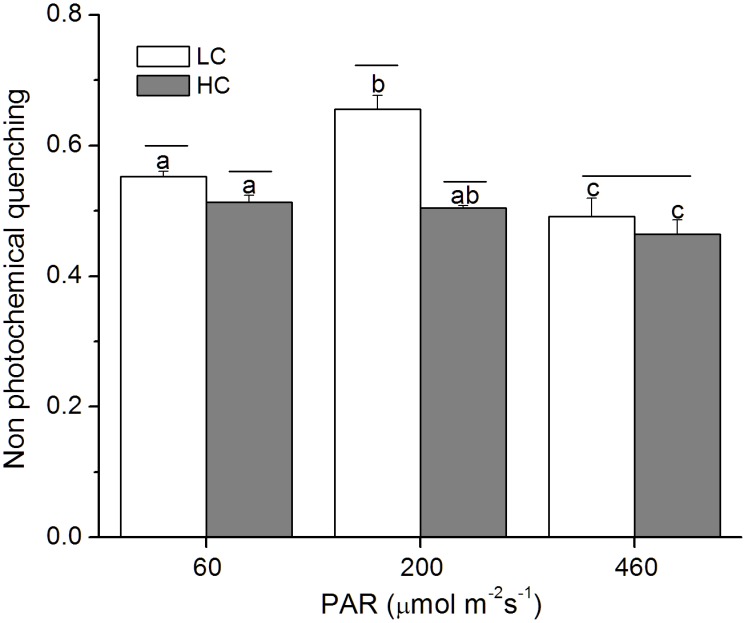
Non-photochemical quenching of *P. tricornutum*. The changes in non-photochemical quenching of *P. tricornutum* cells grown under the LC (390 ppmv, pH 8.18) and HC (1000 ppmv, pH 7.82) and then both acclimated to different light levels (60, 200 and 460 µmol m^−2 ^s^−1^) for 25–36 generations under high actinic light (1228 µmol m^−2 ^s^−1^). Values are means ± SD, n = 3. The short-lines above the histogram bars indicate significant difference between LC and HC, and the different letters indicate significant differences among the light treatments within the HC- or LC-grown cells at *p*<0.05.

The LL-grown cells showed higher non photochemical quenching (NPQ) when exposed to a light stress, which decreased with the increase of growth light levels (Fig. 4). In addition, the NPQ was significantly reduced by the elevated CO_2_ under the low (*p* = 0.006) and medium (*p*<0.01) light levels (Fig. 4).

### Photosynthetic Affinity for Inorganic Carbon

**Figure 5:**
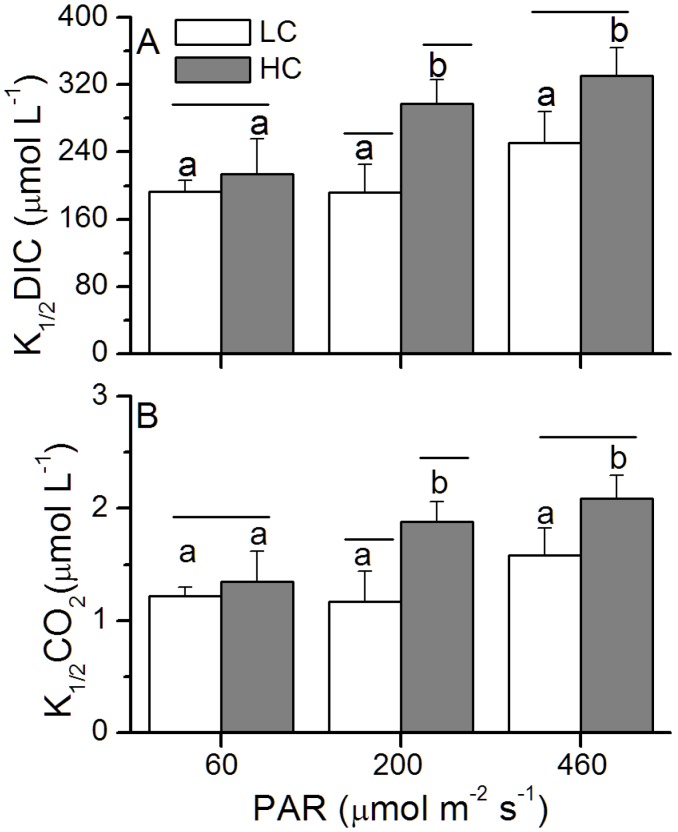
Half-saturation constants (K_1/2_) for DIC (A) and CO_2_ (B) of *P. tricornutum*. Half-saturation constants (K_1/2_) for DIC (A) and CO_2_ (B) of *P. tricornutum* cells grown under the LC (390 ppmv, pH 8.18) and HC (1000 ppmv, pH 7.82) and then both acclimated to different light levels (60, 200 and 460 µmol m^−2 ^s^−1^) for 25–36 generations under middle actinic light (830 µmol m^−2 ^s^−1^). Values are means ± SD, n = 3. The short-lines above the histogram bars indicate significant difference between LC and HC, and the different letters indicate significant differences among the light treatments within the HC- or LC-grown cells at *p*<0.05.

For the HC-grown cells, dissolved inorganic carbon (DIC) or CO_2_ levels for half saturation of photosynthesis (K_1/2_ DIC or K_1/2_ CO_2_) increased by 11%, 55% and 32% under the low, medium and high light levels, respectively, and the differences between the LC and HC was only significant at ML level (*p* = 0.02, Fig. 5), marginally significant at HL (*p* = 0.05), insignificant at LL level (*p* = 0.45). In addition, values of rETR_max_ increased with the increase of growth light levels.

### The Recovery of the Photochemical Yield under Dim Light

**Figure 6:**
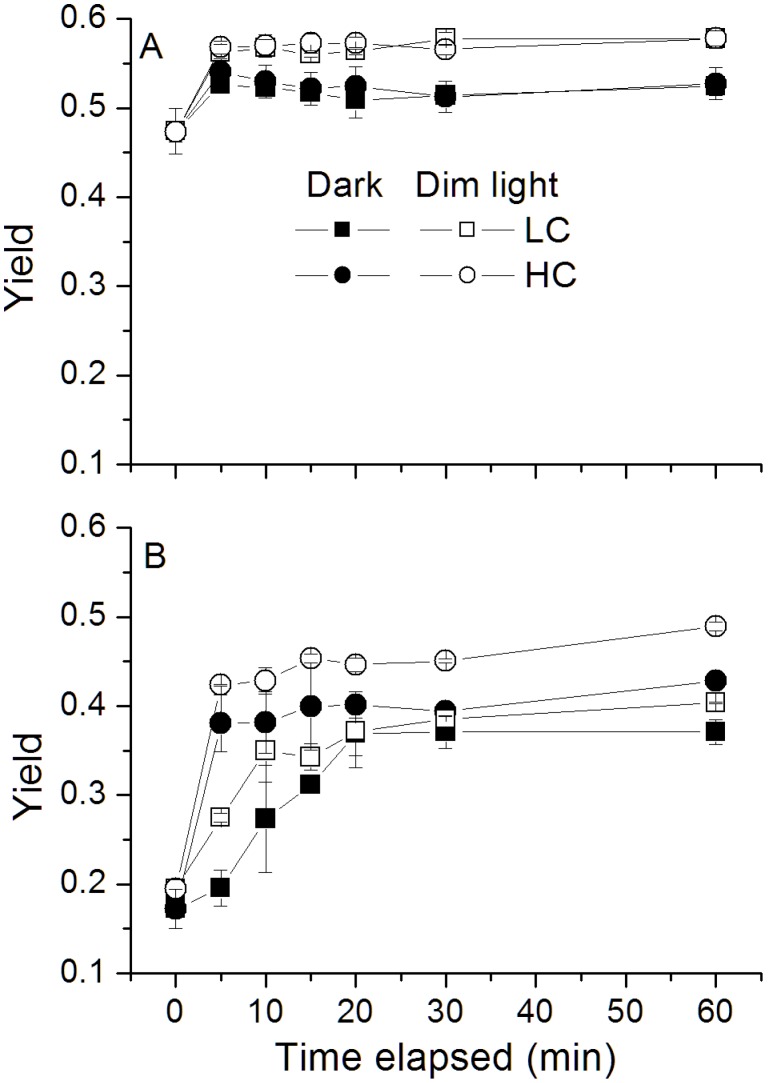
The changes in quantum yield (F_v_/F_m_) of *P. tricornutum*. The changes in quantum yield of *P. tricornutum* cells grown under the LC (390 ppmv, pH 8.18) and HC (1000 ppmv, pH 7.82) and then both acclimated to different light levels (A: 60 µmol m^−2 ^s^−1^; B: 460 µmol m^−2 ^s^−1^) for 25–36 generations under dark or dim light (10 µmol m^−2 ^s^−1^). Values are means ± SD, n = 3.

Under the dark or dim light level, the maximal photochemical yield of PSII, F_v_/F_m_, increased rapidly, reaching the largest value after 10 min, and then remained constant (Fig. 6A, B). Compared with the dark condition, faster recovery of F_v_/F_m_ was observed in the presence of dim light and the cells acclimated to the HC condition showed a higher recovery rate compared to that of the LC-grown ones, especially for the cells grown under HL level (Fig. 6B).

## Discussion

In this study, growth rate of *P. tricornutum* was enhanced by the elevated CO_2_ concentration under photosynthesis-limited, half-saturated and saturated light levels, being consistent with previous studies [11], [12], [36], [37]. Nevertheless, the present study provided the first evidence that the enhanced extent of growth under OA increased with acclimated light levels up to 460 µmol m^−2 ^s^−1^. This result is consistent with our previous results obtained under low sunlight levels, but contradictory to that under high sunlight levels with daytime average PAR over 200 µmol m^−2 ^s^−1^ (Table 4), that led to inhibited growth rate under the OA condition [12]. In the present study, when the cells had been grown at PAR of 460 µmol m^−2 ^s^−1^, their growth rate was still enhanced by the same OA condition by 18% (Fig. 1; Table 4), However, the growth rates were comparatively lower as shown in the precious study under fluctuation sunlight of the same level (daytime average, 460 µmol m^−2 ^s^−1^). Such a discrepancy must be attributed to the difference between the indoor constant and outdoor fluctuating light sources. Under the outdoor fluctuating solar radiation, during a day when the mean PAR level of 200 or 460 µmol m^−2 ^s^−1^, the maximal PAR during noon time could exceed 1000 or 2000 µmol m^−2 ^s^−1^, which caused higher levels of NPQ in the HC- than in the LC- grown cells, indicating that outdoor growth, even with the same level of light dose and with UV radiation screened off, could have brought more light stress to the cells grown under OA conditions [12]. Nevertheless, in the present study, compared to the LC-grown cells, HC-grown cells showed lower NPQ (Fig. 4) and higher F_v_′/F_m_′ even under the HL level (Fig. 3). This hints that compared to those grown fluctuating sunlight, the HC-grown cells under HL spent less energy to cope with light stress and photo-acclimation with daily solar radiation changes, therefore, their growth rates were enhanced by elevated CO_2_. Previously, mitochondrial respiration and carbon fixation were shown to be enhanced in *P. tricornutum* [11] and *Thalassiosira pesudonana* [15] under the same OA condition. Enhanced respiration can supplies more adenosine triphosphate (ATP), its consumption can reduce transmembrane thylakoidal pH gradient (ΔpH), therefore, NPQ could be reduced (Fig. 4). Consequently, the OA might have led to more ATP consumption with decreased NPQ [38].

**Table 4:** Mean specific growth rates of *P. tricornutum*.

PAR (µmol m^−2 ^s^−1^)	Mean specific growth rate (µ; day^−1^)			
	Indoor (constant light level)		Outdoor (solar radiation)	
	LC	HC	LC	HC
60	1.33	1.49 (12%)	0.81	0.95 (17%)
200	1.40	1.59 (14%)	1.01	1.00 (−0.1%)
460	1.21	1.43 (18%)	1.07	0.88 (−18%)

Mean specific growth rates of *P. tricornutum* cells grown under LC and HC conditions and both acclimated (for 25–36 generations) to the constant growth light levels in the present study compared to that observed in the previous study under fluctuating sunlight (acclimated for 21–25 generations) [12]. The data in parentheses represent the percentage change between the LC and HC conditions. Note, the µ values are much smaller under fluctuating than under the constant light regimes.

In addition, a common structural adaptation to light increase involves the reduction in antenna size or the light harvesting ability in order to reduce the absorption of light energy [39], or to increase the carotenoid content to dissipate excessive energy [40]. In our study, compared to LL- or HL-grown cells, the cells grown at medium light level showed higher growth rates, and with increased light level, Chl *a* per cell and the ratio of Chl *c* to Chl *a* sharply declined (Fig. 2A), being consistent with that previously reported in diatoms [41], [42], that altered the proportions of chlorophyll *a*/*c* and chlorophyll *a*/fucoxanthin protein-pigment complexes. The highest ratio between the carotenoids and Chl *a* concentration at the high light level reflects higher capacity of photoprotection (Table 2). Additionally, cells grown at the LL with the lowest initial E_k_ and higher apparent α (Table 3) imply higher light use efficiency. The fact that HC-grown cells showed higher α and lower E_k_ values compared to that of LC-grown ones implies that OA enhanced light use efficiency (Table 3), thus, growth rates were enhanced under the LL and ML levels. The apparent α of HL-grown cells was also enhanced, supporting the enhanced growth rate of the cells under OA, though the absolute specific growth rate was lower than that under LL and ML.

Most diatoms operate CO_2_ concentrating mechanisms (CCMs) [43] to accumulate intracellular CO_2_ and increase the CO_2_/O_2_ ratio around Rubisco [44]. The activity of the enzyme carbonic anhydrase (CA), which accelerates the inter-conversion between HCO_3_^−^ and CO_2_, is known to be down-regulated by elevated CO_2_, leading to a reduction of the active transport or use of HCO_3_^−^ [45]. The HC-grown cells had higher K_1/2_ DIC and K_1/2_ CO_2_ which were increased by 11–55% under the different light levels (Fig. 5), indicating a light dependent down-regulation of CCMs. Given that the operating of CCMs is energetically costly, phytoplankton cells (such as *Chlorella vulgaris*, *Anabaena variabilis*, *Dunaliella tertiolecta*) grown under low light levels usually show a decreased capacity and/or affinity for DIC transport [43], [46]–[51]. Such a down-regulation has been thought to be due to energy limitation under low light [43], [50], [51]. However, in the present study, the CCM of *P. tricornutum* was down-regulated under 1000 ppmv to a less extent in the low light compared to that under medium and high light levels (Fig. 5). Under the photosynthesis-oversaturating light level (HL), the CCM down-regulation was about 3 times that under the limited light level (LL) (Fig. 5). The cells grown at the low light level showed increased Chl *a* content (Fig. 2A) and higher apparent α (Table 3), suggesting that the increased light capture efficiency to meet the energy allocation to CCM operation, especially for the LC-grown cells, which need more energy for operation of CCMs.

Diatoms are subject to photoinactivation of their PSII reaction centers [52], [53], therefore, augmented capacity for their PSII repair is required to maintain photosynthesis [13]. If repair of photodamaged PSII fails to catch up with photoinactivation, PSII would suffer photoinhibition [54]–[56]. OA treatment is known to increase the susceptibility to photoinactivation of PSII [13] and then increased the capacity of PSII repair [19]. Such an increased capacity provides an explanation for the faster recovery of the F_v_/F_m_ under dim light (Fig. 6). Considering dynamic light environment that phytoplankton cells are exposed to, the cells grown under OA conditions with higher recovery rate will suffer less photodamage [19].

With progressive ocean changes, enhanced stratification due to global warming [24], [57] will expose phytoplankton cells in the upper mixing layer (UML) to increased integrated sunlight levels and doses. Such an ocean change may enhance light stress to phytoplankton cells within UML. On the other hand, ocean acidification can result in further light stress for surface phytoplankton assemblages [12]. Therefore, ocean changes due to increased CO_2_ concentration in the atmosphere are likely to trigger higher photoinhibition, though repairing processes in diatoms could be stimulated.

In conclusion, the OA condition under 1000 µatm CO_2_ stimulated the growth of *P. tricornutum* rates under either light limiting or photosynthesis over-saturating light levels, with higher extent of the growth enhancement under the high light level, though the higher specific growth rate was found under the low and medium light levels. The discrepancy between the present and previous study [12] in growth response to OA under fluctuating sunlight could attribute to additional energy requirement for the diatom to cope with light stress and photo-acclimation with diurnal solar changes. Such a hypothetical theory, though requires further experimental testing, is supported by a recent finding in the diatom *Chaetoceros debilis* that showed OA increased cellular quota of particulate organic carbon under constant light levels but decreased it under changing light regimes [58]. This hints that the diatom cells grown under sunlight or fluctuating light regimes may be stressed to allocate more energy for photoprotection and acclimation and resulted less carboxylation and cellular carbon storage.
